# Disciplinary incorporation: Conditions enabling CAM’s expansion and institutionalization in Europe and North America

**DOI:** 10.1177/13634593261462957

**Published:** 2026-06-26

**Authors:** Ayşe Polat

**Affiliations:** 1 Boğaziçi University, İstanbul, Türkiye

**Keywords:** complementary and alternative medicine (CAM), traditional and complementary medicine (T&CM), medical pluralism, WHO policies on CAM, disciplinary incorporation

## Abstract

Drawing on a critical interpretive synthesis of interdisciplinary scholarship, this article examines how complementary and alternative medicine (CAM) expanded and was institutionalized in Europe and North America from the 1970s onward amid broader transformations in medicine, society, and global governance. Contributing to Health’s 30th anniversary special issue, this article identifies six intersecting conditions that collectively enabled CAM’s growing visibility, recognition, and institutional incorporation: (1) intellectual, feminist, and epistemological critiques that destabilized biomedical authority and re-centered lived experience; (2) a broad social turn in public health that reframed disease causation through structural and behavioral determinants; (3) the rethinking of modernization and the emergence of medical pluralism, which challenged universalist accounts of modern medicine; (4) neoliberal restructuring and consumerism, which marketized care and expanded spaces for alternative therapeutics; (5) the scientification and professionalization of CAM through research, clinical trials, and training infrastructures; and (6) the growing influence of the World Health Organization, which promoted CAM’s incorporation into health systems while standardizing practices and conditioning recognition on alignment with biomedical norms. Together, these shifts produced a heterogeneous field shaped by disciplinary incorporation: CAM practices were selectively legitimated insofar as they were rendered legible to biomedical, scientific, and regulatory frameworks, redrawing rather than simply widening the boundaries of medical legitimacy.

## Introduction

Although traditional forms of healing have existed across different cultures and historical periods, their use as alternative or complementary treatments, alongside biomedicine, has been an ongoing site of contestation. These therapies have been named and categorized in diverse ways, giving rise to longstanding debates over how to define and classify Complementary and Alternative Medicine (CAM). Multiple terms are in circulation: traditional medicine (TM), complementary and alternative medicine (CAM), traditional/complementary and alternative medicine (TM/CAM), and traditional and complementary medicine (T&CM). While the World Health Organization (WHO) and many national policies have more recently adopted T&CM—a shift that signals both the decline of “alternative” and a broader, more globally inclusive framing of these practices—this article retains CAM for consistency with the historical period under focus and the academic sources examined. Where specific policy texts, especially those of WHO, employ different terminology, those terms are cited as used in the original documents. Despite recent proposals to define CAM around probable specific effectiveness (Kremling and Schildmann, 2025), definitions remain contested, and the issue is not merely terminological. The labels used for CAM reflect different assumptions about medicine, varying perceptions of the epistemic authority of biomedicine, and transformations within the medical profession and health-care systems. They also shape budgets, guidelines, training, research priorities, and modes of regulation.

Beyond naming and definitional debates, CAM encompasses a heterogeneous set of practices that scholars have variously grouped into subfields—ethnic medical systems, manipulative systems, mind–body therapies, nature-cure therapies, and non-allopathic systems (Fulder, 1996)—or paradigms—body, body–mind, body–energy, and body–spirit (Tataryn, 2002). Despite their diversity, many CAM modalities share a holistic orientation, an emphasis on mind–body–spirit interconnection, a focus on well-being beyond cure, attention to “vitality,” and collaborative healer–patient relations (Goldstein, 1999).

A growing body of research has charted patterns of CAM use, examining who turns to which treatments, under what conditions, and with what expectations. Landmark surveys in high-income countries (Eisenberg et al., 1993; Wiesener et al., 2018) have documented sizeable and durable CAM uptake, with variation by country and modality. More recent analyses show that within Europe, overall use has remained relatively stable in the last decade (2014–2023), while user profiles have been strikingly consistent—women, middle-aged groups, and the highly educated (Kemppainen and Kemppainen, 2025). A systematic review of 231 studies (Tangkiatkumjai et al., 2020) found that expectations of benefit were the most common reason for CAM use (84%), followed by dissatisfaction with conventional medicine (37%) and perceptions of safety (37%). Taken together, these studies document significant and sustained demand for CAM therapies in Europe and North America, particularly from the 1970s onward, even if more recent patterns indicate stabilization rather than continued acceleration.

At the same time, it would be misleading to assume that CAM’s expansion and official recognition pertain only to the West, or that they have unfolded in similar ways even within Europe and North America. There remain major differences across settings in naming, legal status, professionalization, and market size. This article nevertheless focuses on a particular trajectory: the growing visibility, uptake, recognition, and institutionalization of CAM in Europe and North America since the 1970s, and its subsequent global diffusion through research, policy, and governance frameworks. This rise cannot be reduced to a single cultural, economic, or political cause (McQuaide, 2005). Existing scholarship has illuminated many of these dynamics—the decline of biomedical authority, the rise of medical pluralism, the role of neoliberalism, the regulatory ambitions of the WHO—but typically from within particular disciplinary or thematic perspectives. In contributing to Health’s 30th anniversary special issue, this article looks backwards to better understand CAM’s present and future positioning. Reading these strands together, the article traces some of the intersecting intellectual, social, economic, and institutional conditions that helped make CAM’s rise possible. It does not claim that CAM is a recent invention; traditional medicine has existed across cultures for centuries. Rather, it argues that after the 1970s a historically distinctive shift occurred: CAM became more publicly visible, more widely demanded, more formally researched, and increasingly recognized, regulated, and institutionally incorporated in novel ways. This article asks: What conditions made possible this recognition, expansion, and institutionalization of CAM in Europe and North America after the 1970s? And how did shifts in knowledge, economy, governance, and professional practice converge to redraw medicine’s boundaries?

To address these questions, the article draws on a critical interpretive synthesis of the literature (Dixon-Woods et al., 2006). Critical interpretive synthesis is designed for heterogeneous bodies of scholarship where conventional systematic review methods are less suitable. Rather than aggregating findings, it treats synthesis as an interpretive and theory-building process in which concepts and explanatory frameworks emerge inductively through iterative engagement with diverse sources. Accordingly, sources were selected on the basis of conceptual relevance and saturation rather than exhaustive coverage (Perlman et al., 2026). Searches for this article began with broad keywords—including criticism of biomedicine, medical pluralism, medicine and consumerism, World Health Organization and CAM, CAM in the West, and history of CAM and biomedicine—and were subsequently expanded through engagement with additional primary and secondary sources identified during the review. Through iterative reading, comparison, and critical engagement with this interdisciplinary literature—spanning history of medicine, sociology, medical anthropology, postcolonial theory, and global health policy, alongside primary institutional documents—a set of recurring explanatory themes was identified and subsequently consolidated into six analytical transformations.

These transformations are: (1) the destabilization of biomedical authority, driven by intellectual, philosophical, and epistemological critiques that challenged biomedicine’s dominance and sought to humanize medical practice and revalue lived experience; (2) the social turn in public health, which reframed disease causation through behavioral, social, and structural determinants rather than biomedical reductionism; (3) the rethinking of modernization and the rise of medical pluralism within the social sciences, which challenged universalist claims of modern medicine and legitimized diverse epistemologies; (4) neoliberal restructuring and consumerism, which marketized health care, reframed patients as consumers, and created the global economic space for CAM’s commodification and expansion; (5) the scientification and professionalization of CAM, driven by new training programs, research initiatives, and expanding publication infrastructures; and (6) global governance, where WHO promoted CAM’s integration into health systems while simultaneously standardizing practices and setting regulatory benchmarks according to biomedical norms. Together, these transformations form an analytical framework for understanding the diverse conditions that enabled CAM’s institutional expansion in Europe and North America and, subsequently, shaped its wider transnational circulation—a process this article conceptualizes as disciplinary incorporation.

These transformations are not intended to be exhaustive. Rather, they represent the conditions that this article identifies across diverse studies as enabling CAM’s rise and institutionalization. They are not strictly chronological, nor do they imply direct causation. They unfolded in parallel and across overlapping periods: some, such as epistemic critiques of biomedicine and the rethinking of modernization, significantly preceded WHO’s regulatory and governance interventions, while others developed concurrently. They also differed in the directness and intentionality of their relationship to CAM. Earlier intellectual and cultural shifts—critiques of biomedicine, the social turn in public health, the rethinking of modernization—were largely unintended enablers, creating receptive conditions without being designed to advance CAM itself. By contrast, the scientification of CAM and especially WHO’s governance strategies involved more deliberate institutional efforts to define, regulate, and incorporate CAM into health systems. Yet incorporation and disciplining as analytical dynamics were never confined to one set of transformations alone—they ran, with different intensities and logics, through all six. CAM’s expansion was therefore not the product of a coordinated project but of the convergence of forces operating at different levels of intentionality and scale—from deep structural and intellectual shifts to deliberate institutional strategies—which is precisely what makes it such a complex and revealing case.

To address this complexity, this article draws on two theoretical perspectives—Sewell’s (1992) account of social structure and agency and Foucault’s (1980, 1991) analytics of power and governmentality—that together illuminate how CAM’s expansion was both structurally enabled and subjected to disciplinary incorporation.

Sewell’s (1992) conceptualization of social structure and agency refuses to treat structures as rigid and determining, conceiving them as dynamic, plural, and intersecting sets of cultural schemas and resources that are sustained as well as transformed by social interaction. Structures are multiple and operate at different levels; their accumulation is not entirely predictable, they intersect and may overlap with one another, and their reproduction is not automatic (Sewell, 1992). While structures are mutually sustaining, they operate at different levels with different logics, and their intersection can generate outcomes that no single structure individually determined or intended. The six transformations identified in this article embody and illustrate this dynamic: they are distinct social structures that individually and through their convergence produced CAM’s emergence and expansion in the West.

Structures empower and constrain social action and are reproduced by that action; they work through agents who act with and against other structures and apply them to new contexts (Sewell, 1992). The various individual and collective agents involved in CAM’s expansion—intellectual critics, feminist movements, anthropologists, CAM practitioners, patient advocates, WHO bureaucrats and researchers—were not simply carried along by structural currents, whether intentionally or not. They resourcefully exercised differential individual and collective agency that both reproduced and transformed the structural conditions around CAM.

Yet while Sewell recognizes the reciprocal relationship between structures and agents, his framework does not attend to modern disciplinary power as a constitutive force of governance. For Foucault (1980), power is not merely repressive or coercive but productive. It generates knowledge, discourse, and the very categories through which practices become visible, legitimate, or marginal. Governmentality operates not through direct coercion but through the ensemble of “institutions, procedures, analyses and reflections, the calculations and tactics” that allow the exercise of power over populations (Foucault, 1991: 102). Power is relational and diffused, rendering different domains of everyday life governable beyond governments and bureaucratic apparatuses alone (Dean, 1999). The reciprocal constitution of power and knowledge means that governing a domain requires and produces specific forms of knowledge about it (Lemke, 2007). In CAM’s case too, governance operated through the very frameworks of knowledge production that defined what it was, what counted as evidence for its efficacy, and which practices were rendered normal or marginal.

This Foucauldian framework allows us to think beyond both Sewell’s account of structure and agency and DiMaggio and Powell’s (1983) institutional isomorphism—the argument that organizations converge toward dominant institutional forms through coercion, mimicry, or normative pressures. CAM’s convergence toward biomedical forms of clinical and scientific practice, training, and credentialing was not a matter of organizations copying successful models under legitimacy pressures. It was rather a condition of recognition itself: practices were rendered legitimate only insofar as they were first rendered legible to biomedical and bureaucratic frameworks. This article conceptualizes this dynamic as disciplinary incorporation—a simultaneous double movement of incorporation and disciplining in which the disciplining was not external to the incorporation but was its very mechanism.

By incorporation, this article refers to the selective and conditional process through which CAM practices were brought within official health systems, professional infrastructures, and market circuits—granted recognition, but mostly to the extent they were rendered legible to bureaucratic, scientific, and regulatory frameworks set by dominant institutions. By disciplining, it refers to the conditions attached to that incorporation: the defining, classifying, standardizing, and regulating of heterogeneous practices according to norms set by dominant scientific, professional, and governance frameworks (Ashworth and Cloatre, 2022; Carlessi, 2024). Crucially, these two processes were not sequential but simultaneous: recognition was never unconditional, and inclusion was never neutral. What the following sections trace is therefore not simply CAM’s expansion, but the disciplinary incorporation of CAM into health markets and systems in the West—and through it, the redrawing rather than the simple widening of medicine’s boundaries.

The theoretical framework developed here also has its own limitations worth acknowledging. Sewell’s account of multiple intersecting structures illuminates the pluralism and contingency of CAM’s emergence but does not theorize hierarchy among structures—it does not address which structures carry more causal weight in particular conjunctures or why some transformations proved more consequential than others. Foucault’s analytics of governmentality, meanwhile, captures the broad logic of disciplinary incorporation but tends toward a certain totality that can make power appear more coherent and unified than it is in practice. Mapping the conditions behind CAM’s expansion and integration into medicine in the West, and later beyond it, clarifies how shifts in knowledge, economy, and governance collectively reshaped the boundaries of medicine.

## Destabilizing biomedical authority: Intellectual and epistemological conditions for CAM’s rise

The destabilization of biomedicine’s authority became especially visible in the 1970s and 1980s, when diverse intellectual currents advanced critiques that redefined medicine not only as healing but also as a form of social control. These critiques did not directly produce CAM. However, they helped erode the taken-for-granted epistemic and moral supremacy of biomedicine, thereby creating a more receptive cultural and intellectual environment for alternative approaches to health. Zola (1972) coined the term “medicalizing society” to capture how medicine extended into everyday life, while Conrad and Schneider (1980) showed how “medicalization” redefined social problems as medical ones. Illich’s (1975) *Medical Nemesis* deepened the critique, arguing that modern medicine not only manufactured demand for services but also undermined people’s autonomy and capacity for self-care. His notion of “clinical iatrogenesis” offered a powerful indictment of medicine’s unintended consequences.

Epistemological critiques further destabilized biomedicine’s authority. Jewson (1976), drawing on Kuhn, traced historical shifts in medical knowledge production, showing how institutional arrangements privileged doctors’ authority and hospital settings over lay perspectives (Armstrong, 2000). Foucault’s (1963) *Birth of the Clinic* provided a broader lens, analyzing how medical discourses and institutions produced “medicalized subjectivities” through disciplinary power. In different ways, these critiques challenged the assumption that biomedicine alone could define legitimate knowledge about the body, illness, and healing—an important precondition for the wider legitimacy later claimed by CAM.

These critiques converged with the anti-psychiatry movement and feminist scholarship, both of which further questioned biomedical authority and expanded the range of perspectives seen as relevant to health and illness. Cooper’s term “anti-psychiatry” (1967) captured the work of Szasz, who rejected mental illness as a medical category, highlighting its role in social control (Roberts and Itten, 2006). Laing reframed patients’ experiences as meaningful responses rather than pathological deviations, developing what Scott and Thorpe (2006) call “a critical theory of the psychiatric colonization of the lifeworld” (p. 343). Feminist writers and women’s health movements of the 1970s similarly revealed how biomedicine reinforced patriarchal structures—medicalizing childbirth, pathologizing female biology, and naturalizing restrictive gender roles (Turshen, 2020). Their insistence on situated, embodied knowledge opened space for alternative frameworks emphasizing women’s agency, lay knowledge, and holistic perspectives (Scully, 2023).

This matters because CAM’s expansion in Europe and North America did not occur in an intellectual vacuum; these movements legitimized more diverse, individualized, and power-sensitive approaches to health, making CAM more plausible in a context where biomedicine was increasingly criticized for medicalization, epistemic dominance, and insensitivity to lived experience. McQuaide (2005) argues that shrinking civic participation encouraged a privatization of purpose, with individuals turning to personal health and self-care—including alternative medicine—as a new locus of meaning. At the same time, erosion of trust in physicians, dissatisfaction with managed care, awareness of medical errors, and recognition of biomedicine’s limits further drove patients toward alternatives (McQuaide, 2005).

Concurrently, new movements within scientific and clinical paradigms promoted the humanization of medical practice and the reintegration of lived experience into medical reasoning. These developments further weakened biomedicine’s exclusive claim to neutrality and monopoly on truth. Instead of an unquestioned progressive force, biomedicine was increasingly criticized for its narrow conception of health, privileging “scientific objectivity” over lived experience, and reducing patients to biological cases. Postmodern critiques reinforced this shift, advancing narrative and patient-centered approaches. Polkinghorne (1988) described this as a “narrative turn,” in which patient stories were revalued as essential to understanding illness. Kleinman (1978) similarly showed how explanatory models revealed dimensions of suffering invisible to biomedical frameworks. In this context, the so-called “discovery of the patient” emerged: concerns such as patient satisfaction, unmet needs, and the demand for comprehensive care moved to the center of debate.

These shifts overlapped strongly with themes emphasized by many CAM modalities: holism, attention to individual experience, and treatment of the person rather than the disease alone. The biopsychosocial model (Engel, 1977) and the holistic health movement crystallized these connections, emphasizing the integration of psychological, social, and even spiritual dimensions into healing. Critics such as Cassileth and Schwartz lamented that physicians had become “technically proficient but emotionally detached,” losing sight of the patient as a person (Winnick, 2005). At the same time, Freidson (1970) emphasized that by the 1970s medicine’s dominance was increasingly curtailed by state regulation, corporate oversight, and managerial logics. While professional authority persisted, it became fragmented and contested. In the U.S., rising health costs and inadequate insurance produced what was widely described as a “health care crisis” in the 1970s and 1980s, followed by the rise of “managed care” in the 1990s. Corporate logics and cost-containment strategies entered the physician–patient relationship, reshaping professional authority and fueling patient demand for alternatives (Pescosolido and Boyer, 2010).

Together, these developments did not cause CAM’s rise in any simple or intentional sense, but they operated as unintended structural enablers—eroding the epistemic and cultural schemas that had sustained biomedical monopoly and creating the receptive conditions under which CAM could appear to more patients and practitioners as a credible, desirable, or necessary alternative and complement to biomedicine. None of these intellectual currents was designed to advance CAM; their contribution was to pluralize and open the field of legitimate health knowledge, producing an outcome no single one of them individually anticipated. It was upon these opened and pluralized conditions that the later, more deliberate processes of disciplinary incorporation would operate.

## The social turn in public health: From medical causation to social determinants

While the first transformation operated primarily at the level of cultural and epistemological schemas—destabilizing biomedicine’s intellectual authority—the social turn in public health revealed its practical and explanatory limits from within, operating through a different structural logic and at a different institutional level. By the 1970s, new disease patterns and systemic pressures exposed the biomedical model’s limits. The “epidemiologic transition” (Coreil, 2008)—in which chronic conditions such as cancer, heart disease, diabetes, and mental disorders supplanted infectious diseases as the leading causes of morbidity and mortality—revealed the inadequacy of a germ-centered approach. Addressing these conditions required sustained attention not only to biological mechanisms but also to behavioral, social, environmental, and cultural determinants of health. In this sense, the social turn in public health helped widen the boundaries of what counted as relevant to health and illness, thereby creating a context in which CAM’s emphasis on holistic approaches to health, the relationship between lifestyle and well-being, and prevention-oriented approaches could appear more plausible and legitimate.

From the late 1950s onward, the social sciences—including history, demography, medical sociology, medical anthropology, and political science—contributed increasingly to public health (Coreil, 2008). Social historians emphasized that the history of medicine should be read not only through intellectual advances but also through dynamics of social transformation (Cook, 2011). Sociologists and social epidemiologists likewise showed that improvements in population health, such as rising life expectancy, were shaped less by medical advances than by socioeconomic, environmental, and political conditions. The institutionalization of this orientation was marked by the creation of medical sociology as a subsection of the American Sociological Association in 1959. By the 1960s, studies across the United States and Europe—especially Britain—highlighted the social causes of health and disease.

Although social approaches to health have 19th-century roots, systematic attention to non-medical determinants crystallized more clearly from the 1970s onward. A key turning point was the WHO’s 1978 International Conference on Primary Health Care, which placed social determinants at the center of global health policy through the “Health for All by the Year 2000” initiative. Empirical research reinforced this direction. McKeown et al. (1975) showed that mortality decline in the UK long preceded modern medical technologies and was instead attributable to improved nutrition, sanitation, and clean water. Similarly, the UK Black Report—formally the 1980 UK government-commissioned report *Inequalities in Health*, chaired by Sir Douglas Black—documented stark inequalities in mortality linked to income, education, housing, diet, and working conditions (Gray, 1982). Although the UK government largely ignored its conclusions, the Black Report became a landmark in health inequalities research and, together with sociological and epidemiological studies of the 1970s and 1980s, helped consolidate the view that health and illness were fundamentally shaped by social conditions rather than medical advances alone.

The social turn in public health did not directly produce or advocate CAM. However, by displacing strictly biomedical explanations of disease and broadening the range of factors recognized as relevant to health, it prepared the ground for legitimizing different forms of healing. It was driven by its own internal logic—the practical and explanatory limits of the biomedical model in the face of chronic disease and social factors influencing health—and had no stake in CAM’s fortunes. Yet the questions it normalized about lifestyle, environment, social conditions, and holistic well-being were precisely the questions CAM modalities had long claimed as their own. In this sense, it widened the field of what could count as a serious response to illness, without anyone intending that outcome.

## Rethinking modernization and medical pluralism

While the social turn in public health reframed disease causation within societies, broader critiques of modernity and empire redefined medicine’s position within global power dynamics. After World War II, modernization theory emerged as a dominant social-science framework, presenting development as a linear transition from tradition to modernity. Medicine was central to this vision (Gallagher, 1988). Modern health systems were portrayed as rational, bureaucratic, and commodified, embodying progress and symbolizing civilizational advancement—the triumph of science over superstition and of professional doctors over traditional healers (Hayes-Bautista and Minkler, 1979; Turner, 2000). In this narrative, modern medicine stood as both an indicator and a carrier of modernization. These assumptions also contributed to the marginalization of non-biomedical healing traditions, which were often treated as backward residues destined to disappear. Their later revaluation was therefore closely tied to wider critiques of modernization itself.

By the 1970s, these modernist assumptions came under increasing criticism. The dichotomy depicted between tradition and modernity was questioned in multiple ways (Alexander, 1994; Gordon, 1989). The broader postmodern condition, in Lyotard’s sense, further weakened confidence in progress, rationality, and universal truth (Leezenberg, 2017). Postmodern thinkers denied science’s exclusive claim to objectivity and emphasized fragmented, local knowledges. Eisenstadt’s (1999) theory of “multiple modernities” likewise rejected singular developmental trajectories and suggested plural pathways to modernity. Within this shifting intellectual climate, CAM could be reinterpreted less as unscientific and “backward,” and more as a legitimate expression of diverse therapeutic epistemologies, resonating with postmodern valorizations of plurality and difference (McQuaide, 2005). Postcolonial scholarship reinforced this shift, arguing that Western medicine was never a self-contained system but was shaped through colonial encounters and global exchanges (Bhambra, 2011), which further weakened the assumption that biomedicine represented a universal benchmark of progress.

It was within this broader intellectual climate that the comparative analysis of health-care systems gained attraction, with anthropologists playing a leading role in developing the concept of medical pluralism (Baer, 2011). From the 1970s onward, anthropologists systematically analyzed alternative health systems and explicitly critiqued Western medicine (Inhorn and Wentzell, 2012). The institutionalization of medical anthropology as a subsection of the American Anthropological Association in 1972 reflected this recognition. Building on earlier figures such as Benjamin Paul, who in the 1950s stressed the importance of cultural context, this scholarship highlighted biomedicine’s hegemonic claims, bureaucratization, and the alienation it produced in North America and Europe (Good, 1994).

Leslie’s (1976) edited volume *Asian Medical Systems*, based on a 1971 Wenner-Gren symposium, was especially pivotal. Leslie was the first to apply the concept of medical pluralism in a sustained way, showing that while biomedicine claimed exclusivity as the only “scientific” medicine, pluralistic arrangements had long existed in both Western and non-Western contexts (Baer, 2011). His work on India demonstrated how diverse practices persisted despite biomedical dominance. The late 1970s and early 1980s saw a surge of cross-cultural studies. A 1978 Social Science and Medicine special issue featured Leslie’s reflections alongside Kleinman’s influential framework for analyzing health care systems as cultural systems through “explanatory models” (Kleinman, 1978). John Janzen emphasized African, Asian, Central American medical systems as changing social systems (Janzen, 1978a), while Foster and Anderson (1978) and Young (1976) proposed typologies of health systems. Conferences and publications on Chinese medicine, including Kleinman et al.’s (1975) *Medicine in Chinese Cultures*, further expanded the field (Leslie, 1980). Kleinman (1980) continued this line of work through case studies in Taiwan, while Janzen (1978b) did so in Zaire. Leslie (1985) later returned to the theme in another *Social Science and Medicine* special issue, this time also addressing “medical revivalism”—the re-emergence and reinvention of traditional practices in modern contexts (Baer, 2011).

By the mid-1980s, anthropologists increasingly turned their attention inward, examining complementary and heterodox practices within the West. Cassidy et al. (1985) observed that anthropologists had long studied “exotic” systems abroad but largely neglected professionalized alternatives at home, such as naturopathy, homeopathy, and chiropractic. They prepared a bibliography, and this work encouraged more systematic engagement with these therapies and extended the concept of medical pluralism to Western heterodox medicine. This was important for CAM’s later visibility, as it provided an analytical vocabulary through which non-biomedical practices in Europe and North America could be understood not as irrational leftovers, but as part of plural and socially embedded therapeutic landscapes.

At the same time, the concept of pluralism revealed conceptual limits (Khalikova, 2021). Worsley (1982) problematized the category of “non-Western medical systems,” arguing that it homogenized diverse traditions and reflected anthropological assumptions of systematicity. He further questioned whether “medical systems” themselves existed as coherent entities, suggesting that this might reflect anthropologists’ own interpretive frameworks rather than local realities. Even so, these debates helped destabilize the dichotomies of East/West and tradition/modern that underpinned earlier modernization narratives. By situating medicine within postmodern and postcolonial critiques, they dismantled the presumed universality of biomedical knowledge and created conceptual space for recognizing plural epistemologies of health—conditions that helped enable CAM’s later rise.

Medical pluralism became more than a descriptive academic tool: it also served as a political strategy through which states and practitioners contested biomedical hegemony and Eurocentrism (Ernst, 2002), even as these same traditions were gradually absorbed into institutional and scientific frameworks on terms set by dominant biomedical and bureaucratic logics. In postcolonial contexts, traditional medicine was reappropriated both as a nationalist symbol affirming indigenous culture and as a pragmatic complement to biomedicine. While modern medicine often retained symbolic prestige as a marker of modernization, traditional practitioners were widely chosen for their accessibility (Khalikova, 2021; Worsley, 1982). Governments likewise mobilized pluralism to claim cultural legitimacy and geopolitical influence. In Sri Lanka, biomedicine is still colloquially called “English medicine,” reflecting its colonial legacy, while “Sinhala medicine” is valorized as a symbol of national identity (Dew and Liyanagunawardena, 2023: 230). In China, the state deliberately coined the category of “traditional Chinese medicine” in the 1950s to promote its therapeutic system globally, while in India, Ayurveda was reframed as national heritage and deployed in pharmaceutical exports and diplomatic alliance-building (Dew and Liyanagunawardena, 2023: 225). In other words, medical pluralism was a concept formulated largely within Western academia, but it became a resource mobilized across very different contexts—both to contest the colonial legacies of biomedical dominance and the universalist claims of modernization theory and to legitimize diverse therapeutic traditions, including those later recognized as CAM in Europe and North America.

The intellectual and anthropological currents examined here were developed to contest biomedical hegemony, modernization theory, and colonial epistemologies, not to advance CAM. Yet by dismantling the epistemic schemas that had marginalized non-biomedical traditions as backward or irrational, and by providing an analytical vocabulary through which plural therapeutic landscapes could be recognized as legitimate, it added a final layer to the conditions of opening within which CAM’s institutionalization became possible. With this third transformation, the conditions of epistemic openness reach their fullest expression, creating the terrain upon which the more deliberate institutional strategies of disciplinary incorporation examined in the following sections would operate with greatest effect.

## Neoliberalism, consumerism, and global markets

While intellectual and cultural re-evaluations redefined medicine conceptually, the late 1970s also brought a profound political-economic reordering of health systems. From the mid-1970s, fiscal pressures and global market integration reshaped health systems across advanced economies. The 1973–75 oil shock and recession, together with high inflation, produced the most serious economic dislocation since the 1930s. Optimism about expanding equity gave way to a preoccupation with cost control (Whiteford and Nixon, 2000). Comparative health-systems research, once centered on equity, pivoted to efficiency.

By the 1980s, economic upheavals and global integration crystallized into a political turn to neoliberalism—prioritizing market solutions, managerial oversight, and the retrenchment of welfare-state commitments. The World Bank became a central actor in global health, promoting reforms that emphasized privatization, decentralization, and regulatory restructuring to align health sectors with market logics (Mackintosh and Koivusalo, 2005). The result was “market-driven politics”: healthcare was increasingly commodified and reorganized around market principles (Leys, 2001).

Neoliberal globalization intensified this trajectory through the 1990s, extending market rationalities and expanding private medical markets. In this context, Collyer (2004) argues that CAM’s incorporation into market structures was pivotal for its mainstreaming. While orthodox healthcare and CAM alike underwent marketization, for CAM in particular this shift marked a transformation from a “cottage industry” to full-scale commercialization and corporatization worldwide. Over time, traditional medical practices were absorbed into commercial circuits—integrated into pharmaceutical industries, marketed as lifestyle products, and commodified in popular culture (Dew and Liyanagunawardena, 2023).

As health systems became more consumer-oriented, patients were increasingly reframed as active healthcare consumers who promoted, normalized, and expanded CAM—particularly in the United States, where consumer activism also facilitated its commercialization (Goldner, 2004). By the 1990s, CAM had become a substantial market in high-income countries; in the United States alone, annual out-of-pocket expenditures were estimated at roughly $14 billion (Dew and Liyanagunawardena, 2023). This consumer turn intersected with the period’s reflexive individualism: health choices were treated as acts of identity (re)construction (Hughes, 2015).

At the same time, rising health expenditures in the West intensified system pressures and made alternatives such as CAM appear attractive. Although demographic and social factors, including population aging, obesity, and unemployment, also contributed, technological innovation was the dominant driver of spending growth in OECD countries (Nghiem and Connelly, 2017). Against this backdrop, CAM was increasingly presented as a lower-cost, less technology-intensive complement or alternative to pharmaceutical and surgical interventions.

Crucially, despite the global diffusion of neoliberal reforms, CAM’s economic dynamics differ sharply across contexts. In many low- and middle-income countries, CAM is chosen primarily for reasons of affordability and accessibility. Traditional practitioners have often outnumbered biomedical doctors by orders of magnitude—in Africa, for instance, there is roughly one doctor per 40,000 people but one traditional healer per 500 (Dew and Liyanagunawardena, 2023: 222). In such settings, CAM’s persistence or expansion cannot be understood primarily through consumer choice or lifestyle politics; it is also rooted in uneven infrastructure, affordability, and everyday therapeutic necessity. These contrasts serve as a reminder that the neoliberal consumerist dynamics described above are primarily a feature of high-income Western contexts, and that CAM’s global diffusion has taken markedly different forms elsewhere.

The broader economic impact of CAM, however, remains under-evaluated. The heterogeneity of CAM complicates assessment, and some modalities rely on advanced technologies. Systematic reviews indicate that only a minority of therapies demonstrate cost-effectiveness—most notably acupuncture for migraine and manual therapy for neck pain (Herman et al., 2005)—while robust cost–benefit analyses are scarce, and evidence of system-wide savings remains limited (Pelletier et al., 2010).

Neoliberal restructuring thus mattered not only because it expanded markets for CAM, but also because it marked the first transformation in which the disciplinary incorporation dynamic became explicitly visible. Unlike the earlier intellectual and cultural shifts that pluralized the field of legitimate health knowledge without being designed to advance CAM or subject it to institutional governance, neoliberal restructuring both created the economic space for CAM’s expansion and introduced the market and regulatory logics through which that expansion would increasingly be managed. States began to govern therapeutic plurality through regulation, accreditation, and controlled incorporation; CAM was drawn into commercial circuits while simultaneously becoming subject to new forms of oversight. This double movement—expansion and containment, marketization and governance—anticipates the more explicit disciplining dynamics that become fully visible in the scientification and WHO governance transformations that follow.

## The scientification of CAM: Academic uptake and evidence-making

While neoliberal restructuring reframed medicine around markets and consumer choice, parallel transformations unfolded within academia and professional medicine—and unlike the earlier intellectual and cultural shifts that operated as unintended structural enablers, the scientification of CAM marks a shift toward more deliberate institutional pursuit of legitimacy, and with it the beginning of the disciplining dynamic that would define CAM’s incorporation into official health systems. Academic publishing, new training programs, and professional discourse began to recast CAM as a legitimate object of scientific inquiry. The 1980s witnessed a rapid expansion of colleges and formal training programs. In Canada, chiropractic colleges were established in the 1960s, while in the UK, the transition from apprenticeship to formal training occurred in the late 1970s and 1980s. Accreditation and degree status became major goals by the mid-1980s; naturopathy colleges also emerged in the US and Canada around 1978 (Cant and Sharma, 2000). Since then, several universities have opened schools or departments for chiropractic, acupuncture, and naturopathy in both Western and non-Western contexts, and training programs have proliferated in Europe, North America, China, and India. Together with expanding publication venues, these developments positioned CAM within professional education and scientific debate.

Publication patterns also mirror shifting professional stances. Analyzing five leading medical journals, Winnick (2005) identifies three broad phases: condemnation (1960s–70s), reassessment (1970s–early 1990s), and integration (1990s). In the United States, CAM was initially ridiculed as “quackery,” then gradually achieved legal recognition and, in specific domains, reimbursement; acupuncture, for example, entered under tight physician control (Winnick, 2005). By the mid-1990s, consumer demand, the holistic health movement, and national survey evidence of CAM’s popularity (e.g. Eisenberg et al., 1993) pushed sections of the medical establishment away from outright dismissal toward conditional incorporation. The institutionalization of evidence-based medicine reinforced this shift. The U.S. National Institutes of Health established the Office of Alternative Medicine in 1992, signaling entry—however modest—into formal public research funding. By the late 1990s, controlled trials of selected CAM therapies were underway, reframing practices such as acupuncture and herbal medicine as legitimate, if contested, objects of scientific assessment. The term “integrative medicine” gained traction to describe this dual incorporation-and-control dynamic (Winnick, 2005).

Bibliometrics echo this consolidation. Brodin Danell et al. (2020) trace CAM’s emergence as a research domain between 1966 and 2016 through more than 105,000 Medline-indexed publications, documenting a shift from marginal topic to a small but distinct field. CAM’s share of Medline output peaked around 2004–2005 at roughly 0.7%, then flattened after 2011 to about 0.4%–0.5% annually. Growth was geographically differentiated: North America dominated early, acupuncture research became increasingly China-led, yoga by India, and homeopathy by Europe—especially the U.K. and Germany (Brodin Danell et al., 2020). CAM-focused journals consolidated the area while signaling partial exclusion from mainstream outlets; at the same time, CAM studies also appeared in specialty medical and social-science journals (e.g. oncology, gynecology, nursing, sociology, anthropology). Clinical trials rose from under 5% of CAM publications in the early period to roughly 20% in recent years, but often remained small and exploratory, reflecting limited resources and conditional acceptance (Brodin Danell et al., 2020).

These developments also intersected with a broader expansion in the production of health knowledge by international organizations. Surveying intergovernmental bodies between 1950 and 2015, Zapp (2018) shows a marked rise in scientific output from the 1980s, with particular momentum in the 2000s. Bodies such as the OECD—and especially the WHO—became central to global scientific publishing; in this landscape, health, medicine, and pharmaceuticals ranked second only to energy in publication output, and the WHO emerged as a leading producer and coordinator of health-related knowledge (Zapp, 2018).

In short, scientification did not eliminate skepticism, but it redefined CAM as researchable, teachable, and professionally negotiable, and in doing so, it operated as the crucial hinge between the article’s two analytical moments. Unlike the earlier transformations, in which the disciplinary incorporation dynamic remained largely implicit, scientification made it explicit: the pursuit of legitimacy through biomedical frameworks was now a deliberate institutional ambition rather than an unintended structural outcome. Yet in seeking that legitimacy, CAM institutions progressively modeled themselves on biomedical forms of training, research, and publication—converging toward the very standards they had once contested. Once recast as an object of scientific research, CAM could more readily be classified, standardized, and selectively incorporated into official health systems on biomedical terms. This scientific framing also prepared the ground for international organizations—above all the WHO—to codify, regulate, and disseminate CAM at the global level, completing the passage from unintended emergence to deliberate institutional shaping.

## WHO: Promoter and gatekeeper of CAM

If the earlier transformations created the conditions under which CAM could appear plausible and legitimate, the WHO’s governance strategies represent the moment at which those conditions were translated into explicit institutional frameworks—the point where unintended enablement gives way to deliberate incorporation and disciplining on a global scale.

Although the 1948 Constitution defined health broadly, systematic WHO strategies on CAM only began to take shape in the early 21st century. While the 1978 Health for All agenda remained influential, by the 1980s–1990s WHO priorities shifted from equity toward efficiency, aligning with World Bank prescriptions and broader neoliberal policies that emphasized market logics and private-sector partnerships (Mackintosh and Koivusalo, 2005: 188). In this policy climate, CAM was framed not only in cultural terms but also as affordable, accessible, and potentially cost-effective—often pragmatically, to address resource gaps and shortfalls in biomedical provision (Khalikova, 2021).

Analysis of WHO’s policy texts shows that this reframing involved what may be called “disciplining through recognition”: CAM was granted legitimacy, but only insofar as it was rendered legible to bureaucratic, scientific, and regulatory frameworks. This dynamic operated even where recent WHO texts appeared inclusive, as they acknowledged “explicable and non-explicable” knowledge; in practice, the regulatory apparatus tends to cast CAM as a bureaucratic object within national systems.

This disciplining is visible first in WHO’s politics of naming, and second in the regulatory instruments—guidelines, strategies, and benchmarks—through which that naming was operationalized.

### Governance through terminology

Over successive decades, the WHO’s terminological shifts were not merely linguistic adjustments but instruments of governance. WHO shifted from “alternative medicine” in earlier decades to “traditional medicine” in the 2000s and, more recently, to “complementary” and “integrative” medicine in the 2010s. This was more than a terminological shift: each label signaled a regulatory stance and a different degree of legitimacy. “Alternative” marked externality to biomedicine; “traditional” foregrounded cultural embeddedness; and “complementary” or “integrative” implied conditional incorporation within evidence-based and cost-effective frameworks.

In the 1990s and 2000s, overlapping labels (TM, CAM, TM/CAM, T&CM) created shifting boundaries of similarity and difference. Around 2000, WHO sought to systematize usage by associating “traditional medicine” with Africa, Asia, Latin America and “complementary and alternative Medicine” with Europe and North America, while often treating “traditional” and “complementary/alternative” as cognate categories. Over time, “alternative” lost traction, while “complementary” was increasingly embraced and eventually codified in T&CM (World Health Organization (WHO), 2019). Survey evidence also suggested greater user acceptance of “complementary,” though the asymmetry persisted: legitimacy remained conditioned on an adjunct role.

More recently, WHO’s language of “integrative” and “comprehensive” has dissolved explicit oppositions, situating non-biomedical practices as specialized modalities within a unified, patient-centered therapeutic framework. This discursive consolidation tends to recast difference as compatibility: it grants recognition while also neutralizing critique, legitimizing CAM insofar as it can be standardized, regulated, and folded into biomedical systems.

As Carlessi (2024) shows, these terms were mobilized not simply to describe existing practices but to confer legitimacy while sanitizing heterogeneity and aligning practices with biomedical logics. Likewise, labels such as traditional, alternative, complementary, and integrative map ongoing power struggles: “alternative” was sidelined for its oppositional stance, while “complementary” and later “integrative” appear less confrontational yet remain implicitly hierarchical, positioning CAM beneath biomedicine (Polat, 2025). In this sense, naming both legitimizes and disciplines CAM, granting recognition chiefly to forms that can be standardized and rendered commensurable with biomedical norms. This regulatory turn also depoliticized traditional medicine by recasting it as a technical object of governance, privileging laboratory-based criteria of safety and efficacy and marginalizing tacit, oral, familial, or spiritual forms of knowledge (Ashworth and Cloatre, 2022: 493).

### Guidelines and the first global strategy (2000–2005)

In 2000, WHO issued its *General Guidelines for Research and Evaluation of Traditional Medicine*, addressing debates around safety and evidence-based efficacy, while seeking to establish standards for clinical research. Traditional medicine was broadly defined as “the sum total of the knowledge, skills and practices based on the theories, beliefs and experiences indigenous to different cultures, whether explicable or not, used in the maintenance of health, as well as in the prevention, diagnosis, improvement or treatment of physical and mental illnesses” (WHO, 2000: 1). The guidelines outlined three key objectives: (1) developing methodologies for research and evaluation; (2) improving the quality and value of research; and (3) providing appropriate evaluation methods to facilitate regulation and registration (WHO, 2000: 2).

Building on this, the Traditional Medicine Strategy 2002–2005 became WHO’s first comprehensive global policy framework. It called for the integration of traditional and complementary practices into health systems where safe, effective, and affordable; the expansion of research and regulatory oversight; the promotion of rational use; and improved access for poorer populations (WHO, 2002: 43). In framing its priorities, WHO emphasized traditional medicine’s accessibility and affordability in many low-income countries as well as the potential of complementary medicine for managing chronic diseases amid global population aging.

The strategy also sought to document and preserve accumulated knowledge while linking it to modern science through evidence-based research (WHO, 2002: 44). A major outcome was the Global Atlas on Traditional, Complementary, and Alternative Medicine (Bodeker et al., 2005)—the first systematic global survey of policy, regulation, education, research, practice, and use—based on expert meetings in 2001 and 2003. The Atlas became an important reference point for documenting the diversity and governance of traditional and complementary medicine worldwide. In parallel, the strategy countered biomedical critiques that dismissed traditional medicine as irrational (Macartney and Wahlberg, 2014), emphasizing affordability, accessibility, and effectiveness—especially for chronic conditions. This framing was reinforced by Wilfried Kreisel, Director of the WHO Centre for Health Development in Japan, who observed in the Atlas foreword that the 1978 “Health for All” goal remained unrealized—“in practice, health for some”—and traditional medicine’s accessibility was an essential asset for populations facing communicable and non-communicable burdens (Bodeker et al., 2005).

### Professionalization and global benchmarks (2014–2023)

If the 2002–2005 strategy emphasized recognition and incorporation, the 2014–2023 strategy shifted toward promoting regulation by member states and professional standardization. The *WHO Traditional Medicine Strategy 2014–2023* marked a shift toward professionalization of CAM. It prioritized the recognition of practitioner qualifications and the harmonization of educational standards and regulatory frameworks (Goldblatt and Gomes, 2022). As part of this agenda, WHO issued international benchmarks for training programs covering whole systems (e.g. Traditional Chinese Medicine, Ayurveda, Unani, chiropractic, naturopathy, anthroposophic medicine) and specific bodywork therapies (e.g. tui na, nuad thai). The benchmarks set minimum education requirements, defined scopes of practice, and established safety guidelines, thereby embedding CAM within formal professional infrastructures.

### Expansion of national policies and regulation

The 2002–2005 strategy had tangible policy effects. WHO initially aimed to raise the proportion of member states with a national policy on traditional or complementary medicine from 13% to 25% (WHO, 2002: 44), yet the actual figure reached 31% (Bodeker et al., 2005). WHO’s (2019) *Global Report on Traditional and Complementary Medicine* suggests that institutionalization advanced rapidly during the 2010s: 98 countries had adopted a national policy on CAM, 107 had established national CAM offices, and 75 had created national CAM research institutes. Regulatory measures adapted by member states also expanded: 124 countries had regulations on herbal medicines, 78 regulated CAM practitioners, and 45 extended health insurance coverage to CAM services. These developments reflected both policy diffusion and the increasing alignment of national frameworks with WHO benchmarks.

In WHO (2022a) documents, these developments are presented as evidence of progress toward two strategic objectives: expanding scientific knowledge and research on CAM through national policies, and increasing regulation of CAM products, practices, and practitioners in order to ensure their safe and appropriate use. Subsequent WHO (2022b) documents identified further milestones: the inclusion of TM/CAM diagnoses in the 11th revision of the International Classification of Diseases (ICD-11) and the establishment of the WHO Global Centre for Traditional Medicine in India as a hub for research, innovation, and international policy coordination. At the same time, member states reported persistent challenges: limited robust evidence, insufficient funding, weak safety monitoring/pharmacovigilance, and shortages of training and regulatory expertise.

Hoenders et al. (2024)—writing on behalf of national consortia of academic medical centers in the United States, Brazil, the Netherlands, and Germany—credit WHO with advancing institutional recognition but also highlight stark variation: long-standing incorporation in China and Japan, minimal institutional footholds in many African countries, and substantial differences within Europe. Nearly half of WHO member states still lack adequate regulation. Recommendations for the 2025 strategy include greater policy harmonization, expanded research and funding, and stronger international cooperation.

In sum, the WHO has functioned as both promoter and gatekeeper of CAM—the clearest institutional expression of the double movement this article has traced throughout. Through nomenclature, guidelines, and regulatory benchmarks, it advanced CAM’s integration into health systems while simultaneously disciplining that process according to biomedical norms: granting recognition, but only insofar as heterogeneous practices were rendered legible to bureaucratic, scientific, and regulatory frameworks. This is disciplining through recognition in its most deliberate and global form—not primarily the unintended outcome of intersecting structural shifts, as in the earlier transformations, but the most explicit and deliberate exercise of institutional power over the conditions of legitimacy. The outcome is substantial institutionalization—national policies, professional benchmarks, ICD-11 entries, and a global research center—yet what was institutionalized was not CAM in its epistemic plurality but those practices that could be standardized, classified, and incorporated on biomedical terms.

### Limitations

This article has several limitations. As a critical interpretive synthesis, it does not aim to provide an exhaustive or systematic review of all literature on CAM, but rather to identify and interpret recurring conceptual patterns across a heterogeneous body of scholarship. The selection of sources was therefore guided by conceptual relevance and saturation rather than strict inclusion criteria. The analysis also focuses primarily on the historical and institutional conditions that enabled CAM’s expansion, recognition, and institutionalization in Europe and North America. While this focus helps clarify a specific trajectory, it does not permit systematic comparison either across variations within Europe and North America or between these developments and those unfolding in other parts of the world. Nor does it fully capture the heterogeneity of CAM itself: different modalities have followed distinct historical, epistemic, and regulatory paths, and have not been incorporated, professionalized, or regulated in the same way. The institutionalization of acupuncture, for instance, has not necessarily followed the same dynamics as that of homeopathy or other therapies. As a result, the article cannot fully capture how CAM’s institutional development in Europe and North America was connected to, shaped by, or diverged from developments elsewhere, or how modality-specific differences have shaped patterns of recognition and marginalization. The six transformations identified here should therefore be read as an interpretive analytical framework rather than a definitive or globally comprehensive explanation of CAM’s development. Future research could build on this account through more systematic regional comparisons, closer attention to specific modalities and national regulatory histories, and greater engagement with the perspectives of practitioners and patients whose experiences are often filtered through policy and professional discourse.

## Conclusion

Read together, the dynamics traced here suggest that CAM’s trajectory is less a story of “alternative” healing than a lens on broader transformations in medicine and society: the erosion of biomedical authority, the rise of consumer health culture, the neoliberal restructuring of health systems, and the global circulation of knowledge, standards, and regulation. What this trajectory reveals, more precisely, is not simply a contest between biomedicine and its alternatives, but the process of disciplinary incorporation through which the boundaries of medical legitimacy were actively redrawn.

This expansion emerged not from a single coordinated project but from the convergence of six distinct social structures operating at different levels of intentionality and scale. Some operated as unintended structural enablers creating the cultural and epistemic conditions under which CAM could appear plausible and legitimate, while others involved more deliberate institutional efforts translating that plausibility into markets, institutions, and regulatory frameworks. Yet the dynamics of incorporation and disciplining ran, with different intensities and through different mechanisms, through all six, producing an outcome none of them individually determined or intended.

A consistent double movement, which this article has conceptualized as disciplinary incorporation, marks this trajectory: practices were incorporated into official health systems and markets while simultaneously being disciplined according to biomedical norms. The first three transformations created the conditions of epistemic openness; the latter three converted that openness into structured, if uneven, institutional reality—through the markets and consumer demand that made CAM visible, the scientification that made it evaluable and more professionally acceptable; and the governance instruments that made it controllable. Yet institutional recognition throughout proceeded on biomedical terms—through evidence hierarchies, credentialing, standardized training, and regulated scopes of practice. What was legitimized, in other words, was not CAM in its full heterogeneity or diverse pluralistic forms of healing, but those practices that could be rendered legible to dominant scientific and bureaucratic frameworks.

CAM’s expansion has been real but partial, selective, and deeply uneven. Despite growing visibility and institutional legitimation, the evidence base for many CAM therapies remains limited or contested, and significant gaps persist in regulation, accreditation, and public funding. The same forces that enabled CAM’s rise have also defined its limits, producing a field simultaneously more visible and more constrained than accounts of its expansion might suggest. In high-income settings, CAM has often been tied to dissatisfaction with biomedicine, holistic well-being, and consumer choice; in many low- and middle-income settings, it remains indispensable, affordable, and accessible care. The geography of CAM’s institutionalization further reflects wider asymmetries in scientific production, state strategy, and global influence—asymmetries that shaped not only where CAM was incorporated but on whose terms.

The WHO’s evolving terminology—from “alternative” to “complementary” and more recently “integrative”—captures the logic of disciplinary incorporation particularly clearly. These shifts were not merely linguistic, but instruments of governance through which recognition expanded even as heterogeneity was disciplined by drawing the boundaries of legitimate practice closer to biomedical norms. Early WHO strategies emphasized promotion, recognition, and integration, while later ones shifted toward regulation, professionalization, and standardization. Consequently, practices that were often culturally embedded, locally contested, and epistemically plural were progressively recast as technical objects of governance, to be evaluated through standardized criteria of safety, efficacy, and regulation.

CAM’s trajectory therefore does not signal the erosion of biomedical authority so much as its reconfiguration. Medical legitimacy has been widened, but only selectively, through terms of evidence, governance, and professional control that incorporate some therapeutic forms while marginalizing others. Seen in this light, CAM is not simply an “alternative” to medicine, but a revealing site through which the changing boundaries of medicine itself can be traced. The expansion of medical legitimacy and the narrowing of epistemic plurality are not opposing tendencies but two sides of the same process: the boundaries of medicine were not simply widened—they were redrawn.
